# Do-Not-Resuscitate Orders in Patients With Acute Hypoxemic Respiratory Failure Due to COVID-19: Practices, Predictors, and Outcomes in a Retrospective Study

**DOI:** 10.7759/cureus.93626

**Published:** 2025-10-01

**Authors:** Hasan M Al-Dorzi, Lama Alzahrani, Manar Abaalkhail, Lama Algaraini, Aljohara Bensarhan, Wesal Alharbi, Maha Sadat Gundroo

**Affiliations:** 1 Intensive Care Unit, King Saud bin Abdulaziz University for Health Sciences College of Medicine, Riyadh, SAU; 2 Intensive Care Unit, Acharya Shri Chander College of Medical Sciences, Jammu, IND

**Keywords:** covid-19, dnr order, do-not-resuscitate, end-of-life care, intensive care

## Abstract

Background

Do-Not-Resuscitate (DNR) orders were commonly used in patients with severe Coronavirus Disease 2019 (COVID-19), with variation across different healthcare settings. We evaluated the practices of DNR orders in patients with severe COVID-19 at a tertiary care hospital in Saudi Arabia.

Methods

This retrospective cohort study evaluated patients with acute hypoxemic respiratory failure due to COVID-19 who required ICU admission in Riyadh, Saudi Arabia, between March and December 2020. We compared patients with DNR orders during ICU stay to those without and described their clinical condition on the day of DNR order implementation.

Results

Among 470 patients (median age 62.0 years, 348 (74.0%) were males, 207 (44.0%) used high-flow nasal cannula and/or noninvasive ventilation without intubation, and 263 (56.0%) needed intubation), 30.6% (N = 144) received DNR orders after a median of 10 days in the ICU. Patients with DNR orders were older and had more comorbidities. On the DNR order day, 126 patients (87.5%) were on invasive mechanical ventilation (median fraction of inspired oxygen, 0.80), 84 (58.3%) were on vasopressors, and 42 (29.2%) were on renal replacement therapy. The median Sequential Organ Failure Assessment (SOFA) score was 13. On multivariable logistic regression analysis, age (odds ratio (OR) per one-year increase, 1.052) and intubation (OR, 3.757) were associated with DNR orders. The hospital mortality rate was significantly higher in patients with DNR orders (133/144 (92.4%) vs. 61/326 (18.7%), p < 0.0001).

Conclusions

DNR orders were commonly used in patients with severe COVID-19 during the first wave of the pandemic, typically after one week of ICU admission. Key predictors were older age and persistent organ failure.

## Introduction

The Coronavirus Disease 2019 (COVID-19) pandemic was characterized by an overwhelming influx of patients suffering from severe illnesses due to SARS-CoV-2 infection, leading to an immense strain on healthcare systems worldwide [1,2]. Healthcare providers grappled with the formidable task of providing care to large numbers of patients in the presence of relatively limited bed capacity and resources [1,2]. Some believed that triaging - which patients should receive more advanced care - was needed [2]. The utilization of Do-Not-Resuscitate (DNR) orders was one of the ways to implement rationing of healthcare resources [3,4]. Additionally, early in the pandemic, there were concerns about viral spread during cardiopulmonary resuscitation, thus jeopardizing healthcare providers [5,6]. Moreover, the high mortality rate among patients with severe COVID-19 led many to believe that cardiopulmonary resuscitation might be futile [7,8]. A multicenter study conducted in the United States found that only 48 out of 400 patients with COVID-19 who had cardiac arrest and underwent cardiopulmonary resuscitation (12.0%) survived until hospital discharge; 28 patients (7.0%) had normal or mildly impaired neurological status [8].

Decisions on DNR orders are usually based on a combination of factors that include the patient’s wishes and preferences, as well as his/her medical condition. Furthermore, practices of DNR orders are affected by ethnicity, as well as religious, and cultural values and vary between institutions, countries, and even within the same country [9,10]. One international study found that high-income countries had a significantly higher rate of decisions to withhold life-sustaining treatment (48%) compared to upper-middle-income countries (27%) and low/lower-middle-income countries (20%) among hospital nonsurvivors [10]. Patients with DNR orders in high-income countries were older, had higher illness severity, and more organ support than patients in low/middle-income countries [10]. Additionally, DNR orders were less prevalent in the Middle East (29 out of 354 patients (8.2%)) compared with other countries (1,259 out of 9,524 patients (13.2%) in the whole cohort), despite higher illness severity [10].

At the start of the COVID-19 pandemic, some clinicians advocated for the widespread application of unilateral DNR orders for patients with COVID-19 (without the agreement of patients or their surrogates) [11,12]. This stirred ethical dilemmas regarding the conflict between respecting patient autonomy and ensuring justice and equitable allocation of resources, and posed several medical challenges [11]. Understanding of DNR order practices in different countries remains important, even though the COVID-19 pandemic has ended, as the possibility of future infectious pandemics persists. In Saudi Arabia, practices of DNR orders are affected by a complex interplay between multiple factors, which include Islamic principles and unique cultural values. As a result, these practices may differ from those in other countries, making it essential to explore and understand them. The objective of this study was to describe the implementation of DNR orders in patients with severe COVID-19 early during the pandemic in Riyadh, Saudi Arabia; identify the clinical factors influencing these decisions; and evaluate their associated outcomes, including hospital mortality (primary outcome), length of stay in the ICU and hospital, and duration of mechanical ventilation (secondary outcomes).

## Materials and methods

Setting and patients

This retrospective cohort study was conducted at the Intensive Care Department of King Abdulaziz Medical City, a tertiary care hospital in Riyadh, Saudi Arabia, with more than one thousand beds. The Intensive Care Department had seven different ICU units. In the early waves of the COVID-19 pandemic, four ICUs were assigned to COVID-19 patients [13]. These units were covered by board-certified intensivists providing continuous coverage 24/7. Multidisciplinary clinical rounds were performed daily. Regular family visits to ICU patients were restricted during the pandemic, and families were updated by phone and videoconferencing [13]. The hospital's DNR order policy, which was established well before the COVID-19 pandemic, specified that a DNR order was a purely medical decision, requiring agreement among three qualified physicians before it could be implemented [14]. It also specified that the decision should be justified and documented in the hospital’s electronic medical record [14]. In cases of disagreement with the medical team, full medical support was typically continued. The hospital had an Ethics Committee to resolve any conflict between physicians and the patient/family. As per the pre-existing hospital policy, patients who had DNR orders and were in the Emergency Department or the hospital ward, and were not already on organ support, were deemed not eligible for ICU admission. This study was approved by the Institutional Review Board of the Ministry of National Guard - Health Affairs. Informed consent from patients was waived due to the nature of the study.

This study included all consecutive adult patients with acute hypoxemic respiratory failure due to COVID-19 infection who required ICU admission from March 1 to December 31, 2020. Exclusion criteria were: admission to cardiac ICUs, which were covered by the Department of Cardiac Sciences and did not admit patients with confirmed COVID-19 during the pandemic; and no requirement for oxygen or use of only simple oxygen (nasal cannula or face mask), which usually indicated a nonsevere illness and good prognosis.

Data collection

Medical records were de-identified for data collection. The collected data included demographics and comorbidities, including hypertension, diabetes, obesity (defined as body mass index (BMI) > 30 kg/m²), chronic cardiac, respiratory, kidney, and neurologic diseases, and active cancer (solid or hematologic). Additionally, laboratory results were noted, including white blood cell, neutrophil, and lymphocyte counts; neutrophil-to-lymphocyte ratio; hemoglobin; platelet count; creatinine; lactate; ferritin; fibrinogen; D-dimer; interleukin-6; and chest X-ray findings. We also recorded selected treatments, such as the modality of respiratory support for acute hypoxemic respiratory failure (high-flow nasal cannula, noninvasive mechanical ventilation, invasive mechanical ventilation, and ventilator settings on the first day for intubated patients); renal replacement therapy (continuous or intermittent); and systemic corticosteroid use.

For patients with DNR orders, we also recorded certain key variables, including the date of the DNR order and the clinical status on the day of the DNR order (Sequential Organ Failure Assessment (SOFA) score [15]; use of vasopressors; modality of respiratory support; the highest inspired oxygen fraction (FiO₂); the lowest partial pressure of oxygen in arterial blood to the inspired oxygen fraction (PaO₂:FiO₂) ratio; serum creatinine; and use of renal replacement therapy). We also reviewed the records of patients without DNR orders for documented discussions regarding goals of care and noted instances where the patient or family refused DNR orders.

In this study, the primary outcome was hospital mortality. The secondary outcomes were length of stay in the ICU and hospital and duration of mechanical ventilation.

Data analysis

We classified eligible patients into two groups: those with DNR orders while in the ICU and those without such orders. We reported categorical variables as frequencies with corresponding percentages, and continuous numerical variables as medians along with their interquartile range (IQR). For between-group comparisons, we applied the Chi-square test or Fisher’s exact test for categorical variables, and the Student t-test or Mann-Whitney U test, as appropriate, for continuous variables. We also compared patients with early DNR orders (within three days of ICU admission) to those who had later DNR orders.

To identify the clinical predictors of DNR orders while minimizing model complexity and preventing overfitting, we conducted stepwise multivariable logistic regression analysis, applying backward elimination based on the likelihood ratio test. The independent variables entered into the model were clinical factors that could affect the decision to initiate a DNR order and may contribute to the severity of illness and mortality. These variables included demographics (age, sex, and BMI) and baseline clinical characteristics: preexisting chronic comorbidities (hypertension, diabetes, obesity, chronic cardiac, respiratory, kidney, and neurologic diseases), active cancer, early vasopressor use, receiving invasive ventilation, and laboratory results at ICU admission (white blood cell, neutrophil, and lymphocyte counts; neutrophil-to-lymphocyte ratio; hemoglobin; platelet count; creatinine; lactate; ferritin; fibrinogen; D-dimer; and interleukin-6). In the model, we imputed missing values for continuous variables using the respective medians, as many variables were skewed. The model was also run without variables with a high number of missing data. We reported the variables that remained significant, presenting their odds ratios (ORs) with the corresponding 95% confidence intervals (CIs). A test was considered significant if the two-tailed p-value was <0.05.

All analyses were conducted using IBM SPSS Statistics for Windows, Version 21 (Released 2012; IBM Corp., Armonk, NY, USA).

## Results

Baseline characteristics

We studied 470 adults who required admission to the ICU for acute hypoxemic respiratory failure due to COVID-19. The baseline characteristics of the study patients are summarized in Table 1. The median age was 62.0 years (IQR 51.0, 72.0), and the majority were male (n = 348; 74.0%). Preexisting comorbidities were common in the study patients, especially diabetes (n = 294; 62.6%), hypertension (n = 277; 58.9%), and obesity (n = 233; 49.6%).

**Table 1:** Baseline characteristics of patients *Student t test; **Mann-Whitney U test; ***Chi-square test The number of patients with missing results was as follows: BMI (n = 2), white blood cell count (n = 3), neutrophil count (n = 81), lymphocyte count (n = 80), hemoglobin (n = 3), platelet count (n = 8), creatinine (n = 2), lactate (n = 55), fibrinogen (n = 236), D-dimer (n = 80), ferritin (n = 45), and interleukin-6 (n = 455). BMI: body mass index, DNR: do-not-resuscitate, IQR: interquartile range

Variable	All patients (N = 470)	DNR (N = 144)	No DNR (N = 326)	Test value	p-value
Age (years), median (IQR)	62.0 (51.0, 72.0)	71.0 (59.3, 78.0)	59.5 (48.0, 69.0)	7.3*	<0.0001
Male sex, N (%)	348 (74.0)	101 (70.1)	247 (75.8)	1.6***	0.20
Female sex, N (%)	122 (26.0)	43 (29.9)	79 (24.2)	1.6***	0.20
BMI (kg/m^2^), median (IQR)	30.0 (25.8, 34.2)	29.4 (26.0, 33.2)	30.1 (25.6, 34.4)	-0.5**	0.60
Comorbidities, N (%)					
Obesity, N (%)	233 (49.6)	67 (46.5)	166 (50.9)	0.8***	0.38
Hypertension, N (%)	277 (58.9)	96 (66.7)	181 (55.5)	5.1***	0.02
Diabetes, N (%)	294 (62.6)	99 (68.8)	195 (59.8)	3.4***	0.07
Chronic cardiac diseases, N (%)	62 (13.2)	20 (13.9)	42 (12.9)	0.1***	0.77
Chronic respiratory disease, N (%)	70 (14.9)	23 (16.0)	47 (14.4)	0.2***	0.66
Chronic kidney disease, N (%)	42 (8.9)	22 (15.3)	20 (6.1)	10.3***	0.001
Chronic neurologic disease, N (%)	27 (5.7)	17 (11.8)	10 (3.1)	14.1***	<0.0001
Active cancer	7 (1.5)	4 (2.8)	3 (0.9)	2.3***	0.13
Laboratory findings, median (IQR)					
White blood cell count (x10^9^/L)	9.5 (7.0, 13.0)	10.0 (7.0, 12.6)	9.2 (7.0, 13.0)	0.4*	0.72
Neutrophil count (x10^9^/L)	7.4 (4.9, 10.3)	7.7 (5.3, 10.1)	7.2 (4.8, 10.5)	-0.1**	0.90
Lymphocyte count (x10^9^/L)	0.9 (0.6, 1.4)	0.8 (0.6, 1.2)	1.0 (0.7, 1.4)	-2.6**	0.01
Neutrophil-to-lymphocyte ratio	7.6 (4.0, 12.9)	9.0 (4.7, 14.3)	7.0 (4.0, 12.1)	-2.0**	0.04
Hemoglobin (g/L)	130 (116, 142)	126 (109, 136)	132 (117, 144)	-3.0*	0.003
Platelets (x10^9^/L)	252 (193, 330)	249 (192, 312)	256 (193, 333)	-0.5*	0.60
Creatinine (µmol/L)	84 (70, 119)	87 (73, 137)	82 (68, 115)	-2.1**	0.03
Lactate (mmol/L)	1.8 (1.4, 2.5)	1.9 (1.4, 2.7)	1.8 (1.4, 2.5)	-0.7**	0.48
Fibrinogen (g/L)	5.2 (4.1, 6.7)	5.1 (3.8, 6.3)	5.2 (4.1, 6.9)	-0.7**	0.50
D-Dimer (μg/mL)	1.4 (0.8, 3.6)	1.9 (1.0, 4.0)	1.2 (0.7, 3.0)	-2.9**	0.004
Ferritin (ng/mL)	714 (330, 1683)	699 (382, 1593)	718 (314, 1823)	-0.2**	0.86
Interleukin-6 (pg/mL)	86 (21, 108)	86 (32, 3683)	82 (18, 100)	-0.4**	0.71
Unilateral infiltrates on chest X-ray	22 (4.7)	9 (6.3)	13 (4.0)	1.1**	0.28
Bilateral infiltrates on chest X-ray	448 (95.3)	135 (93.8)	313 (96.0)	1.1**	0.28

Key management interventions are described in Table 2. A total of 207 patients (44.0%) were treated using high-flow nasal oxygen and/or noninvasive ventilation without intubation, while 263 patients (56.0%) received invasive mechanical ventilation. The ventilator settings on the first day of intubation included a median FiO₂ of 50% and a positive end-expiratory pressure of 12 cm H₂O. Additionally, 147 patients (31.3%) had prone positioning while intubated for severe acute respiratory distress syndrome. Corticosteroids were administered to 380 patients (80.9%).

**Table 2:** Management of patients in the intensive care unit *Student t test; ***Chi-square test For patients on invasive ventilation, the number of patients with missing results was as follows: FiO_2_ (n = 15), PEEP (n = 23), and tidal volume (n = 51). DNR: do-not-resuscitate, FiO_2_: fraction of inspired oxygen, IQR: interquartile range, PEEP: positive end-expiratory pressure

Variable	All patients (N = 470)	DNR (N = 144)	No DNR (N = 326)	Test value	p-value
Systemic corticosteroids, N (%)	380 (80.9)	125 (86.8)	255 (78.2)	4.8***	0.03
Prone positioning, N (%)	147 (31.3)	51 (35.4)	96 (29.4)	1.7***	0.20
High-flow nasal oxygen, N (%)	335 (71.3)	87 (60.4)	248 (76.1)	12.0***	0.001
Non-invasive ventilation, N (%)	255 (54.3)	89 (61.8)	166 (50.9)	4.8***	0.03
High-flow nasal oxygen or non-invasive ventilation without intubation, N (%)	207 (44.0)	31 (21.5)	176 (54.0)	42.7***	<0.0001
Intubation, N (%)	263 (56.0)	113 (78.5)	150 (46.0)	42.7***	<0.0001
Invasive ventilation settings on Day 1, median (IQR)					
Lowest FiO_2_ (%)	50 (40, 60)	50 (40, 65)	45 (40, 60)	1.1*	0.28
Maximum PEEP (cm H_2_O)	12 (10, 14)	12 (10, 14)	12 (10, 14)	-1.6*	0.12
Maximum tidal volume (mL)	400 (360, 450)	400 (360, 450)	400 (360, 450)	-0.9*	0.36
Vasopressors in the first 3 days, N (%)	62 (13.2)	27 (18.8)	35 (10.7)	5.6***	0.02
Renal replacement therapy, N (%)	83 (17.7)	37 (25.7)	46 (14.1)	9.2***	0.002

**Figure 1:**
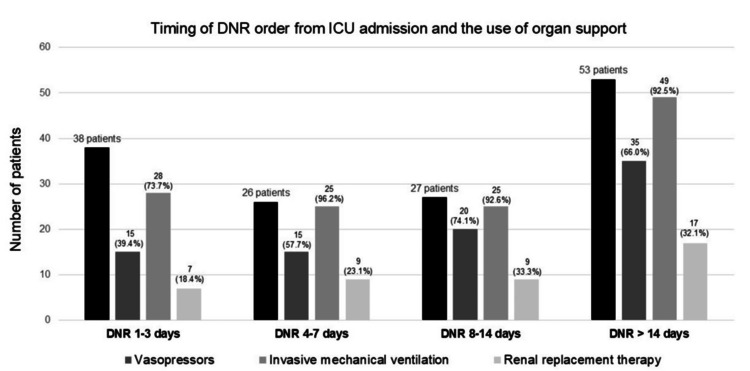
Timing of DNR order from admission to the ICU and the frequency of organ support The black bars represent the number of patients with DNR orders initiated within the specified time period after ICU admission. Numbers in parentheses above the bars represent the percentage of patients with the specified organ support. DNR: do-not-resuscitate; ICU: intensive care unit

The DNR order was implemented in 144 patients (30.6%) at a median of 10 days after ICU admission (IQR 3, 18), which corresponded to a median of 15 days after hospital admission (IQR 5, 22). Our review did not identify any cases in which a DNR order was implemented despite refusal by the patient or their family, commonly defined as a unilateral DNR order. Figure 1 describes the timing of DNR orders from ICU admission and shows that most occurred after Day 3, with 53 patients (36.8%) receiving them after 14 days.

Predictors of DNR orders

Patients with DNR orders were older (median age 71.0 years (IQR 59.3, 78.0) versus 59.5 years (IQR 48.0, 69.0); p < 0.0001) and had a higher prevalence of hypertension, chronic kidney disease, and chronic neurologic disease (Table 1). They were more likely to receive invasive mechanical ventilation (n = 113/144 (78.5%) versus 150/326 (46.0%); p < 0.0001).

On multivariable logistic regression analysis for the clinical predictors of DNR orders, the variables that remained in the model were age (OR per one-year increase, 1.052; 95% CI, 1.034-1.070) and receiving invasive mechanical ventilation (OR, 3.757; 95% CI, 2.330-6.057). The risk remained the same when the variables with high numbers of missing values (ferritin, D-dimer, lactate, fibrinogen, and interleukin-6) were removed from the model.

On the day of DNR order implementation, 126 patients (87.5%) were on invasive mechanical ventilation, four (2.8%) on high-flow nasal oxygen, and 14 (9.7%) on noninvasive ventilation. The median FiO₂ was 80% (IQR 55, 100), and the PO₂:FiO₂ ratio was 91 (IQR 61, 150). Eighty-four patients (58.3%) were on vasopressors - 73 on norepinephrine infusion, with a median dose of 0.25 mcg/kg/min (IQR 0.10, 0.48), and 20 on vasopressin infusion - and 42 (29.2%) were on renal replacement therapy, with a median SOFA score of 13 (IQR 11, 16).

The 38 patients with early DNR orders (within three days of admission) were older compared to patients with later DNR orders, with a median age of 78.0 years (IQR 72.0, 84.3) compared to 68.5 years (IQR 57.0, 76.3; p < 0.0001). Additionally, they had a higher prevalence of chronic respiratory disease (11/38, 28.9%) compared to 12/106 (11.3%; p = 0.01). Compared with patients who had DNR orders after three days in the ICU, those with early DNR orders tended to have less organ support during ICU stay - invasive mechanical ventilation in 15/38 (39.5%) versus 70/106 (66.0%; p = 0.004), and renal replacement therapy in 7/38 (18.4%) versus 35/106 (33.0%; p = 0.09) (Figure 1).

Outcomes

Table 3 presents the key outcomes of the study patients. Overall, 194 patients (41.3%) died in the hospital, with the majority of deaths (171 patients) occurring in the ICU. The mortality of patients with DNR orders was significantly higher than that of other patients (133/144 patients (92.4%) versus 61/326 patients (18.7%), p < 0.0001). Patients with early DNR orders had similar hospital mortality compared with those who had late orders (35 out of 38 (92.1%) and 98 out of 106 patients (92.5%), respectively; p = 0.95). Among the 61 patients who died without a DNR order, the treating physicians documented that the family refused a DNR order in only two patients (3.3%).

**Table 3:** Outcomes of patients **Mann-Whitney U test; ***Chi-square test The number of patients with missing results was as follows: length of stay in ICU (n = 2) and duration of mechanical ventilation (n = 33). DNR: do-not-resuscitate; ICU: intensive care unit; IQR: interquartile range; MV: mechanical ventilation

Variable	All patients (N = 470)	DNR (N = 144)	No DNR (N = 326)	Test value	p-value
Tracheostomy, N (%)	62 (13.2)	26 (18.1)	36 (11.0)	4.3***	0.04
ICU mortality, N (%)	171 (36.4)	114 (79.2)	57 (17.5)	164.2***	<0.0001
Hospital mortality, N (%)	194 (41.3)	133 (92.4)	61 (18.7)	223.5***	<0.0001
Length of stay in ICU (days), median (IQR)	9 (4, 18)	13 (6, 23)	8 (4, 14)	-4.5**	<0.0001
Length of stay in hospital (days), median (IQR)	17 (11, 28)	22 (13, 31)	17 (11, 27)	-2.7**	0.01
Duration of MV in intubated patients (days), median (IQR)	11 (5, 19)	12 (7, 21)	9 (4, 16)	-3.9**	<0.0001

The patients with DNR orders had a longer duration of mechanical ventilation and longer stays in the ICU and hospital.

## Discussion

This study found that nearly one-third of patients who developed acute hypoxemic respiratory failure due to COVID-19 had DNR orders early in the COVID-19 pandemic, at a tertiary-care hospital in Riyadh, Saudi Arabia. These orders were predominantly introduced late in the ICU stay, with most patients requiring substantial organ support. Older age and the requirement for invasive mechanical ventilation served as markers of greater severity of illness among patients with COVID-19 who later had DNR orders. More than 90% of patients with DNR orders (133/144 patients) did not survive hospitalization.

The DNR order is an important end-of-life care decision that has irreversible consequences. It may be justifiable or viewed by the patients and/or physicians as appropriate in the presence of severe chronic health conditions, diminished quality of life, terminal illness, or imminent death, where cardiopulmonary resuscitation is unlikely to yield meaningful clinical benefit. Reaching a DNR decision is a complex process that is affected by multiple factors, including ethics, laws, and policies, and involves prognostication and communication between healthcare providers and patients and/or families [16]. This process is frequently associated with significant moral distress for families and healthcare providers [17]. Early in the COVID-19 pandemic, the DNR decision-making process became more complex, as healthcare systems were strained, physical visits were disallowed or restricted, and communication between healthcare providers and families was limited [1].

We found that 30.4% of the 470 study patients with acute hypoxemic respiratory failure due to COVID-19 had DNR orders. In a previous study in the same ICU, 3,217 out of 24,790 patients (13%), admitted over 19 years (1999-2017), had DNR orders while in the ICU [18]. The rate was higher (18.3%) in the 14,531 patients who were intubated [18]. This suggests that DNR orders were practiced more commonly in patients with COVID-19 and acute hypoxemic respiratory failure. This high rate has been observed in other studies involving patients with COVID-19. For instance, among 1,270 patients admitted to two hospitals in New Jersey between March and May 2020, 860 (67.7%) had a DNR order [19]. Additionally, a large study of adult patients who required ICU admission at two academic hospitals in Chicago found that 271 out of 559 COVID-19 patients (48.5%) had DNR orders [20].

In the current study, the main clinical factors associated with DNR orders were age and multiorgan failure, especially the requirement of invasive mechanical ventilation. These factors have been linked to poor outcomes in COVID-19 [21-23]. Older age is often accompanied by chronic comorbidities and frailty, which together increase the risk of severe illness and higher mortality [22,24]. Invasive mechanical ventilation has been associated with a high mortality rate in patients with COVID-19, reaching approximately 45% in two meta-analyses [21,25]. This mortality rate increased with age, reaching 71.3% in patients aged 61-70 years, 77.1% in those aged 71-80 years, and 84.4% in patients aged >80 years [21].

In the current study, the timing of DNR orders was relatively late in most patients. The 38 patients with early DNR orders (within three days of ICU admission) were older patients with more chronic illnesses. This aligns with findings from other studies, where DNR orders were utilized more frequently in older patients, especially during COVID-19 surges [26]. This may be related, at least in part, to patients’ preferences on life support and end-of-life care. The optimal timing for implementing DNR orders in the adult ICU remains uncertain. In a study of 188 non-bedridden patients with cancer who received invasive mechanical ventilation and had at least one other organ failure, organ failure scores were more predictive of survival on Day 6 than at admission or on Day 3 [27]. Another study suggested that an intensive care trial for one to four days may be beneficial for patients with solid tumors carrying a poor prognosis [28]. A longer trial may benefit patients with hematologic malignancies [28]. Additionally, patients with DNR orders had longer duration of mechanical ventilation and extended stays in the ICU and hospital, compared to those without DNR orders. These findings suggest that they had multiorgan failure and remained dependent on organ support. The late implementation of DNR orders may reflect the failure of high-intensity therapeutic interventions and was probably the outcome of clinical deliberation. 

The hospital mortality rate in our study was high (194/470, 41.3%), which is close to the rates reported in other studies for patients with acute hypoxemic respiratory failure [21,29]. Patients with DNR orders had a higher hospital mortality, reaching 92.4% (133/144), compared to 18.7% (61/326) in those without DNR orders. Most of the patients who had DNR orders received advanced interventions, such as invasive mechanical ventilation and renal replacement therapy, and had a prolonged stay in the ICU. This suggests that the DNR decision was likely not influenced by a lack of resources. Whether family visit restrictions affect the rate and/or timing of DNR order implementation is unclear. A study at two centers in the United States suggested that a restricted family visit policy during the COVID-19 pandemic was associated with delayed implementation of DNR orders and extended ICU stays [30].

This study has limitations. It was a retrospective cohort study conducted at a single center in Riyadh in 2020, during the initial phase of the COVID-19 pandemic and before the widespread availability of vaccines and the development of targeted therapies. Hence, our findings may have limited generalizability to other settings and to the later phases of the pandemic, when clinical practices and patient outcomes evolved significantly. We did not have data about baseline functional status and did not evaluate the patients who had cardiac arrest in the ICU and survived until hospital discharge. Restricted visitation during the study period, changes in communication methods between families and patients, as well as between families and healthcare providers, opinions of physicians on DNR orders, and family dynamics are potential confounders. These factors may have affected our results, but could not be assessed in our study. We also did not review the details of the notes surrounding DNR decisions, but we evaluated the severity of illness and organ support on the day of the DNR order. The high number of patients admitted to the ICUs during the study period may have limited the documentation of clinical data, including conversations about goals of care, in the medical records. The association between DNR orders and invasive mechanical ventilation should not be interpreted as causal. It likely reflects illness severity, which may have influenced the decision to implement a DNR order.

## Conclusions

During the early phase of the COVID-19 pandemic, DNR orders were implemented in nearly one-third of critically ill patients with acute hypoxemic respiratory failure due to COVID-19 at a tertiary-care hospital in Riyadh, Saudi Arabia. Older age and the use of invasive mechanical ventilation were significantly associated with DNR orders, likely reflecting greater illness severity. The implementation of DNR orders was mostly late in the course of the ICU stay, and patients with DNR orders had longer stays in the ICU and hospital, as well as prolonged mechanical ventilation, compared to those without. These findings suggest that DNR orders were generally implemented with clinical deliberation in our ICU early during the COVID-19 pandemic and were unlikely to be used to save resources. More than 90% of patients with DNR orders did not survive hospitalization, reflecting disease severity and probably underscoring the appropriateness of these decisions. Our study also highlights the importance of earlier discussions on the goals of care, particularly in high-risk patient populations. Further research is needed to determine the optimal timing for end-of-life discussions and DNR order implementation in critically ill patients. Given the complexity of these discussions, evaluating the impact of family visitation restrictions during pandemics on the patient care process and outcomes is essential.
